# Emotion regulation and perceptions of academic stress as key predictors of academic motivation in second language learning

**DOI:** 10.1371/journal.pone.0327071

**Published:** 2025-08-18

**Authors:** Haiying Liang, Xu Mao

**Affiliations:** 1 Postdoctoral Researcher, School of Foreign Languages, Peking University, Beijing, China; 2 Lecturer, School of Health Humanities, Peking University, Beijing, China; University of Tartu, ESTONIA

## Abstract

Motivation, as a driving force of human behavior, is influenced by various psychological factors. Among these, emotion regulation and perceptions of academic stress have received limited attention in the context of second language learning. This study examines the interplay between emotion regulation, perceptions of academic stress, and academic motivation among university students in China who are learning English as a second language. A convenience sample of 1189 university students in China participated in the study. Data were collected using validated emotion regulation, perceptions of academic stress, and academic motivation questionnaires and analyzed using Pearson correlation and multiple regression methods. The results reveal that emotion regulation is strongly and positively correlated with academic motivations (r = 0.73, p < 0.01), while perceptions of academic stress have a strong negative correlation with academic motivations (r = −0.75, p < 0.01). Multiple regression analysis shows that emotion regulation significantly predicts academic motivations (B = 0.442, p < 0.01), explaining 44.2% of the variance, with cognitive reappraisal having the strongest positive effect (B = 0.435, p < 0.01). Conversely, perceptions of academic stress negatively predict academic motivations (B = −0.503, p < 0.01), with academic workload and exams exerting the most substantial negative impact (B = −0.492, p < 0.01). These findings highlight the critical role of emotional regulation in enhancing academic motivation and the detrimental effects of academic stress, particularly related to self-perception, on students’ motivation.

## Introduction

Academic motivation plays a central role in second language acquisition, influencing learners’ engagement, persistence, and achievement. Compared to general academic subjects, second language (L2) learning places unique demands on learners by requiring not only cognitive processing but also the use of a non-native language for self-expression, social interaction, and formal assessment [1]. Specifically, L2 learners must simultaneously manage cognitive, linguistic, and affective challenges, which can increase the likelihood of emotional dysregulation [2,3]. In such emotionally charged settings, the capacity to regulate stress and emotions becomes especially critical. Therefore, exploring emotion regulation and perceptions of academic stress in L2 learning offers unique insights into how learners sustain motivation in the face of these distinct pressures. The present study seeks to examine how learners’ emotion regulation strategies and perceptions of academic stress predict their motivation in the specific context of L2 learning. By doing so, this research aims to contribute to a more nuanced understanding of how affective factors shape motivational outcomes in L2 learners, with potential implications for pedagogy and learner support.

### Language learning motivation

Motivation is a key focus in numerous academic fields, where it is understood as a complex construct involving various dimensions such as goals, beliefs, values, emotions, and personal needs [4,5]. Academic motivation plays a critical role in shaping students’ engagement, persistence, and success in learning, particularly in the context of second language acquisition [4]. It encompasses a wide range of psychological components, such as goals, beliefs, emotions, and needs, which collectively drive learners’ behavior and academic performance [5]. Motivation is particularly important in second language learning, as it influences learners’ ability to persist through challenges and sustain their effort over time [6]. For instance, intrinsic motivation can foster a deeper engagement in language practice by sparking interest and enjoyment, while extrinsic motivation, such as achieving academic or career goals, can provide a strong sense of purpose and direction [7].

Previous studies have demonstrated a strong link between motivation and positive learning results. For instance, Liu et al. [8] found that students’ basic psychological needs directly and indirectly (via boredom and motivation) influenced their engagement in learning English. Wang et al. [9] observed that rural-urban-migrant students had lower motivation and engagement compared to their peers, with motivation having less impact on their learning behaviors. Wang and Liu [10] reported that motivation factors influenced intrinsic and extrinsic motivation, both positively affecting English learning behaviors, though intrinsic motivation had a stronger effect. Noels et al [11] found that highly motivated English as a L2 learners tend to achieve better proficiency and exhibit greater persistence in language acquisition. While there has been substantial research on factors predicting academic motivation, such as self-efficacy and goal-setting strategies [12], limited attention has been given to the role of emotional regulation and perceptions of academic stress as predictors of motivation. Understanding these relationships is crucial for developing strategies that support learners in navigating the complexities of second language acquisition and achieving their goals. This study aims to fill the existing gaps in the literature by investigating the predictive roles of emotion regulation and perceptions of academic stress in influencing academic motivation among second language learners. The findings will contribute to a deeper understanding of these psychological factors and provide actionable insights for enhancing second language learning outcomes.

### Emotion regulation

Emotion regulation refers to the various strategies individuals use to influence their emotional experiences, whether by altering the intensity, duration, or expression of their emotions in response to specific situations [13]. Two widely studied emotion regulation strategies are cognitive reappraisal and expressive suppression [14]. Cognitive reappraisal involves reframing situations that evoke strong emotions, such as stress or anger, by adopting a more positive or alternative perspective. In contrast, expressive suppression entails deliberately concealing emotional expressions to align with social or situational norms. While suppression may manage outward reactions, it can lead to a disconnect between internal emotions and external expressions, especially in high-stress scenarios [14,15].

Building on self-determination theory, emotion regulation may serve as a predictor of academic motivation, particularly in the context of language learning. Effective emotion regulation strategies enable learners to navigate and manage the negative emotions frequently associated with language learning challenges, such as anxiety, frustration, and self-doubt. By mitigating the impact of these emotions, learners can maintain a more positive outlook and sustained effort, which are critical for achieving long-term goals in second language acquisition [16].

For example, strategies like cognitive reappraisal can help learners reinterpret setbacks as opportunities for growth, fostering resilience and enhancing intrinsic motivation [17]. Similarly, managing negative emotions effectively can reduce the likelihood of disengagement or burnout, enabling learners to stay focused on their academic or personal goals [18]. In this way, emotion regulation not only fosters a more emotionally supportive context for learning but also strengthens the psychological mechanisms underlying motivation, such as autonomy, competence, and relatedness, which are central to self-determination theory [16].

Previous research has explored the relationships among emotion regulation, academic motivation, and language learning outcomes (e.g., [15,19–22]). However, studies specifically focusing on L2 learners in the Chinese context remain limited. Therefore, this study aims to address this gap by investigating how emotion regulation and perceived academic stress influence motivation among Chinese university students learning English as a second language.

### Perceptions of academic stress

In academic settings, academic stress refers to the pressures students face when academic demands feel overwhelming or difficult to manage, either through personal coping strategies or external support [23,24]. This type of stress arises from various sources, including coursework, exams, grading systems, deadlines, and the expectations set by instructors or peers [25]. Academic stress can have numerous negative impacts on students’ well-being, academic performance, and mental health. Prolonged exposure to academic stress is associated with heightened anxiety, depression, and burnout, which can undermine students’ ability to concentrate and perform effectively [26]. It may also affect physical health by disrupting sleep patterns, weakening the immune system, and increasing the risk of stress-related illnesses [27,28].

Previous research has highlighted the dynamic interplay between stress and academic outcomes, suggesting that while moderate stress can sometimes act as a motivator, excessive or chronic stress typically leads to declines in both performance and well-being [29,30]. Despite these insights, the relationship between perceptions of academic stress and academic motivation remains unexplored. Examining this relationship could offer educators valuable knowledge about how students’ perceptions of academic stress impact their academic motivations and inform the creation of targeted strategies to foster motivation, ultimately aiding students in achieving better academic outcomes.

### Theoretical foundation

Self-Determination Theory (SDT, [31,32]) offers a comprehensive framework for understanding human motivation in learning contexts. According to SDT, motivation can be conceptualized along a continuum ranging from amotivation to extrinsic motivation and intrinsic motivation, with the degree of self-determination increasing from low to high. Central to SDT are three basic psychological needs—autonomy, competence, and relatedness—which, when satisfied, enhance intrinsic motivation and well-being. SDT also emphasizes the role of contextual and individual-level factors in shaping motivational outcomes. Emotion regulation, as a self-regulatory capacity, helps learners manage negative affect and maintain a sense of control (autonomy) and competence in demanding learning situations. Conversely, academic stress may undermine the satisfaction of psychological needs by threatening learners’ sense of efficacy and emotional stability. Thus, the present study adopts SDT as a guiding framework to examine how learners’ ability to regulate emotions and their perceptions of academic stress predict their academic motivation in L2 learning.

### The present study

Building on the theoretical foundation of SDT [31,32], the present study aimed to investigate how learners’ capacity to regulate emotions and their perceptions of academic stress influence academic motivation in the context of English as a L2 learning. Although previous research has explored connections between emotion regulation, stress, and motivation in general academic settings (e.g., [21]), there is a need to contextualize these relationships within L2 learning, where the affective demands are particularly pronounced. In L2 classrooms—especially those that emphasize speaking and active participation—learners often face heightened vulnerability to negative emotions and stress [33]. This makes affective regulation and perceptions of challenge particularly relevant to motivational outcomes.

Therefore, this study examines the predictive power of two psychological factors—emotion regulation and academic stress—on the academic motivation of Chinese university students studying English as a second language. Based on the above rationale, the following hypotheses were proposed:

• Hypothesis 1. Students’ emotion regulation predicts their academic motivations.
• Hypothesis 2. Students’ perceptions of academic stress predict their academic motivations.

### Methodology

This research was approved by the Peking University Institutional Review Board (Approval Number: IRB00001052–25022). Written informed consent was obtained from all participants prior to their participation in the study. Data collection was conducted anonymously to ensure the confidentiality and privacy of participants. The study strictly adhered to the ethical guidelines of academic research integrity, and all necessary measures were taken to minimize potential risks and protect participants’ rights throughout the research process.

### Participants

This study involved a total of 1189 Chinese English as a Foreign Language learners, ranging in age from 18 to 27 years (M = 21.8, SD = 1.68). The sample included 446 male and 704 female students, while 39 participants did not disclose their gender. Participants were recruited using social media channels, primarily WeChat, to facilitate widespread outreach. All participants were native Chinese speakers and had typically been studying English for approximately eight to eleven years. Prior to participation, informed consent was obtained from each individual, after which they were invited to complete the online survey.

### Instruments

The questionnaire used in this study comprised four sections. The first section collected demographic information (e.g., age, gender, year of study), while the remaining three sections measured the key psychological constructs of interest: emotion regulation, academic stress, and academic motivation. All scales employed have demonstrated good psychometric properties in prior research and were adapted appropriately for the L2 learning context.

**Emotion Regulation* *Scale,** created by Gross and John in 2003 [13], consists of 10 questions designed to assess two different strategies for regulating emotions: cognitive reappraisal (6 items) and expressive suppression (4 items). This tool was selected because it focuses on how individuals use these strategies to manage emotions in different settings, such as second language learning. Participants were asked to indicate their level of agreement with each statement using a 5-point Likert scale, with responses ranging from 1 (‘strongly disagree’) to 5 (‘strongly agree’). This instrument has been widely used in educational psychology contexts and shows robust reliability and construct validity (e.g., Gross and John, 2003). In the present study, the internal consistency was acceptable, with Cronbach’s α = 0.927. Confirmatory factor analysis was performed, and the model demonstrated a good fit to the data (χ²/df = 1.86, RMSEA = 0.042, SRMR = 0.028, CFI = 0.975, and TLI = 0.970).

*Perceptions of Academic Stress Scale,* developed by Bedewy and Gabriel in 2015, was used to assess how participants perceive academic stressors [34]. This scale contains 18 questions, organized into three main categories that capture common academic stress sources: academic expectations (4 items), workload and exams (8 items), and self-assessment of academic abilities (6 items). Participants rated their agreement with the stress perception statements on a 5-point Likert scale, where 1 indicates ‘extremely irrelevant’ and 5 indicates ‘strongly relevant’. To minimize response biases, the scoring for five of the items was reversed. The scale has demonstrated satisfactory validity and reliability in prior studies [34]. To reduce semantic redundancy, two items were removed from the original 18-item Perception of Academic Stress Scale. Specifically, item 7 (“Teachers have unrealistic expectations of me”) was excluded due to its overlap with item 6, which also reflects pressure from teachers. Similarly, item 11 (“I believe that the amount of work assignments is too much”) was removed because of its conceptual similarity to item 10, which addresses excessive curriculum workload more broadly. The final version of the scale thus contains 16 items. In our sample, the scale showed good internal consistency (Cronbach’s α = 0.962). A confirmatory factor analysis was conducted and demonstrated an acceptable fit (χ²/df = 2.04, RMSEA = 0.045, SRMR = 0.031, CFI = 0.964, and TLI = 0.956).

*The Academic Motivation Scale*, created by Vallerand et al. in 1992, is a well-established tool used to evaluate academic motivation [35]. It includes 28 items that measure motivation in three core areas: intrinsic motivation, extrinsic motivation, and amotivation. From the original 28-item Academic Motivation Scale, three items were removed to reduce redundancy. Item 6 (“For the pleasure I experience while surpassing myself in learning English”) was conceptually similar to item 13, which more clearly emphasizes personal accomplishment. Item 20 (“To show myself that I am an intelligent person capable of mastering English”) overlapped with other items focused on self-validation, such as item 7. Item 21 (“In order to have a better salary that English skills can help me achieve”) duplicated content found in items addressing career-related external goals (e.g., item 1 and item 8). These deletions resulted in a refined 25-item scale. Although the Academic Motivation Scale is based on a multidimensional model of motivation, we used a composite score of academic motivation in the current study. This approach was supported by prior research suggesting that, in some educational contexts, the subscales of the Academic Motivation Scale may form a second-order general motivation factor [36,37]. A principal components analysis in our sample revealed a dominant general factor explaining the majority of variance across items, and the internal consistency of the total scale was high (Cronbach’s α = 0.977). Therefore, we treated academic motivation as a unidimensional outcome variable representing learners’ general motivation level in the context of L2 learning. The results showed acceptable model fit (χ²/df = 2.14, RMSEA = 0.049, SRMR = 0.035, CFI = 0.961, and TLI = 0.954).

### Data collection

At the outset of the study, instructors from 11 universities in China were contacted and invited to participate. Once the instructors agreed to participate, the researchers sent them consent forms along with an online survey to distribute to their students. The students were informed that their responses would be kept confidential and utilized exclusively for the purposes of the study. The survey links were shared with the students via the widely used WeChat application, which ensured ease of access and participation. Participants were encouraged to complete the questionnaires at their convenience. The data collection process spanned over one month. In the first round (15 April to 15 May 2025), 1,230 responses were collected, of which 1152 were valid. In the second round, conducted over three days in July 2025, 40 additional responses from another university in China were collected, with 37 deemed valid. In total, 1270 questionnaires were collected, and 1,189 were included in the final analysis. Questionnaires were excluded for reasons such as incomplete responses, inconsistent answers, or failure to meet the required criteria for participation, ensuring that only valid and reliable data were included in the analysis.

### Data analysis

The data were analyzed using SPSS software (version 30). Descriptive statistics were first calculated to summarize the central tendencies and dispersion of the main variables: Emotion Regulation, Perceptions of Academic Stress, and Academic Motivation (Table 1). Normality of the data was assessed using Skewness and Kurtosis values. Pearson correlation analysis was then conducted to examine the relationships between these variables (Table 2). This provided insights into the strength and direction of their associations, all of which were tested for statistical significance at the p < 0.01 level. Finally, multiple regression analysis was performed to evaluate the predictive effects of Emotion Regulation and Perceptions of Academic Stress on Academic Motivation (Table 3). Subscales of both predictors were included to explore their specific contributions.

**Table 1:** Descriptive statistics.

Variables	Mean	SD	MIN	MAX	N
Emotion regulation	28.43	5.77	11.0	48.0	1189
Perceptions of academic stress	59.19	10.65	19.0	89.0	1189
Academic motivations	79.44	17.2	32.0	136.0	1189

**Table 2:** Correlation matrix between research variables.

Variables	Emotion regulation	Perceptions of academic stress	Academic motivations	AVE	√AVE
Emotion Regulation	1.000	–	–	0.602	0.776
Perceptions of Academic Stress	−0.65	1.000	–	0.605	0.778
Academic Motivations	0.73	−0.75	1.000	0.618	0.786

Note. AVE = Average Variance Extracted; √AVE = Square Root of the AVE. AVE represents the amount of variance captured by a construct in relation to the variance due to measurement error. √AVE values are typically compared with inter-construct correlations to assess discriminant validity [38].

**Table 3:** Multiple regression analysis.

	Predictors	B (Unstd.)	SE	β (Std.)	R	R²	t	p	VIF	Tolerance
Total score predictor	Emotion Regulation	0.442	0.023	0.415	0.818	0.669	18.912	< 0.01	1.724	0.58
Sub-dimension predictor	Cognitive reappraisal	0.435	0.041	0.419	0.73	0.532	10.644	< 0.01	3.935	0.254
	Expressive suppression	−0.341	0.04	−0.336	0.73	0.532	−8.532	< 0.01	3.935	0.254
Total score predictor	Perceptions of Academic Stress	−0.503	0.023	−0.485	0.818	0.669	−22.096	< 0.01	1.724	0.58
Sub-dimension predictor	Academic expectations	−0.136	0.037	−0.145	0.755	0.569	−3.691	< 0.01	4.233	0.236
	Academic workload and exams	−0.492	0.025	−0.481	0.755	0.569	−19.68	< 0.01	3.78	0.265
	Students’ academic self-perception	−0.155	0.031	−0.156	0.755	0.569	−5.0	< 0.01	3.765	0.266

Note. B = Unstandardized coefficient; SE = Standard error; β = Standardized coefficient; R² = Coefficient of determination; t = t-statistic; p = significance level; VIF = Variance inflation factor; Tolerance = 1/ VIF.

## Results

### Descriptive statistics

The descriptive statistics for the main variables are presented in Table 1. The mean score for Emotion Regulation was 28.43 (SD = 5.77), with scores ranging from 11.0 to 48.0 Perceptions of Academic Stress had a mean score of 59.19 (SD = 10.65), with a minimum score of 19.0 and a maximum score of 89.0. Academic Motivation had a mean score of 79.44 (SD = 17.2), with scores ranging from 32.0 to 136.0.

### Correlation analysis

The correlation matrix in Table 2 highlights the relationships among the key variables in this study. A strong and significant positive correlation was found between Emotion Regulation and Academic Motivations (r = 0.73, p < 0.01), indicating that higher levels of Emotion Regulation are associated with greater Academic Motivation.

In contrast, Perceptions of Academic Stress showed a strong and significant negative correlation with Academic Motivations (r = −0.75, p < 0.01). This finding suggests that as perceptions of academic stress increase, academic motivation tends to decrease significantly.

Additionally, a strong negative correlation was observed between Emotion Regulation and Perceptions of Academic Stress (r = −0.65, p < 0.01), indicating that individuals with higher levels of emotion regulation experience lower levels of academic stress. These results emphasize the interconnected nature of emotion regulation, stress, and motivation in academic contexts.

The AVE values for Emotion Regulation, Academic Stress, and Academic Motivations were 0.602, 0.605, and 0.618 respectively. The square roots of the AVEs (√AVE = 0.776, 0.778, and 0.786) exceeded the corresponding inter-construct correlations, indicating satisfactory discriminant validity in accordance with Fornell and Larcker’s (1981) criterion.

### Multiple regression analysis

To examine the predictive power of emotion regulation and perceptions of academic stress on academic motivation, a series of multiple regression analyses were conducted using both total scale scores and sub-dimensions. Table 3 presents the results of the multiple regression analysis for predicting academic motivation based on Emotional Regulation and Perceptions of Academic Stress. The findings highlight several key relationships:

1. Total Scale Predictors

The total score of Emotion Regulation significantly and positively predicted academic motivation, B = 0.442, SE = 0.023, β = 0.415, t(1189) = 18.912, p < .01. The model explained 66.9% of the variance in academic motivation (R² = 0.669). Multicollinearity was not a concern (VIF = 1.724; Tolerance = 0.580). Similarly, the total score of Perceptions of Academic Stress was a significant negative predictor of academic motivation, B = −0.503, SE = 0.023, β = −0.485, t(1189) = −22.096, p < .01, accounting for 66.9% of the variance (R² = 0.669; VIF = 1.724; Tolerance = 0.580).

1. Sub-Dimension Predictors

When disaggregating the predictors, cognitive reappraisal was found to be a significant positive predictor of academic motivation (B = 0.435, SE = 0.041, β = 0.419, t(1189) = 10.644, p < .01). In contrast, expressive suppression was a negative predictor, albeit with a slightly smaller effect size (B = −0.341, SE = 0.040, β = −0.336, t(1189) = −8.532, p < .01). The subscale model explained 53.2% of the variance (R² = 0.532), with acceptable levels of multicollinearity (VIFs < 5).

Regarding academic stress, all three sub-dimensions were significant negative predictors of academic motivation. Academic expectations had a modest effect (B = −0.136, SE = 0.037, β = −0.145, t(1189) = −3.691, p < .01). In contrast, academic workload and exams exerted a stronger influence (B = −0.492, SE = 0.025, β = −0.481, t(1189) = −19.68, p < .01). Students’ academic self-perception also contributed negatively (B = −0.155, SE = 0.031, β = −0.156, t(1189) = −5.00, p < .01). The combined subscale model explained 56.9% of the variance in academic motivation (R² = 0.569), with acceptable levels of multicollinearity (VIFs < 5).

## Discussion

The findings of this study provide significant insights into the factors influencing academic motivation, particularly the roles of emotion regulation and perceptions of academic stress. These results align with existing literature on the predictors of academic motivation and contribute to a deeper understanding of how these factors interact in educational settings.

The strong positive relationship between emotion regulation and academic motivation suggests that better emotional regulation directly enhances academic motivation. This implies that interventions aimed at improving students’ emotional regulation skills could have a substantial impact on their motivation levels. This result is consistent with earlier studies that highlight the crucial impact of emotion regulation strategies on academic success (e.g., [15,39]), as well as their role in promoting positive emotional experiences, including enjoyment (e.g., [40]).

The positive relationship between cognitive reappraisal and academic motivation observed in this study corroborates prior research highlighting the importance of emotional regulation in educational outcomes (e.g., [13,41]). Moreover, this study’s findings support Self-Determination Theory, which identifies competence, autonomy, and relatedness as key determinants of academic motivation [31]. Cognitive reappraisal enhances students’ perceptions of competence and autonomy, allowing them to tackle academic challenges more effectively and maintain their motivation over time.

The weak negative correlation observed between emotion regulation suppression strategies and academic motivation suggests that the use of emotion suppression as a coping mechanism may hinder students’ ability to stay motivated in their academic pursuits. This finding aligns with previous research indicating that suppression, as a less adaptive emotion regulation strategy, is associated with lower psychological well-being and reduced academic performance [42]. Students who frequently suppress their emotions may experience heightened stress and reduced self-efficacy, both of which are critical for sustaining academic motivation [43,44]. Additionally, the energy spent on managing internal conflicts and emotional regulation may detract from their focus on academic goals.

The weak effect size also suggests that while emotion suppression has a negative impact, it may not be the primary determinant of academic motivations. Other factors, such as positive emotional regulation strategies (e.g., cognitive reappraisal) or external influences (e.g., supportive learning environments), may play a more significant role in driving academic motivations.

The negative relationship between perceptions of academic stress and academic motivation aligns with existing literature that underscores the detrimental impact of excessive academic stress on students’ psychological well-being and motivation. Research by Lazarus and Folkman [45] has long established that stress can be a significant source of emotional strain, affecting individuals’ ability to function effectively, especially when the stress is perceived as unmanageable. Similarly, Pekrun et al. [46] have emphasized that academic stress, particularly when related to perceived academic demands and challenges, can hinder academic performance.

In the current study, the perceptions of academic stress associated with academic expectations and self-perceptions were found to be significant negative predictors of academic motivation. These findings echo previous studies that have suggested that stress related to academic expectations—such as pressure to meet high academic standards or the fear of failure—can erode students’ motivation to engage in their studies [46]. When students perceive academic expectations as unrealistic or unattainable, they may experience feelings of helplessness or inadequacy, which can reduce their enthusiasm and commitment to learning.

Moreover, stress related to self-perception of academic abilities also emerged as a key predictor of decreased motivation in this study. This aligns with the work of Bandura [47], who highlighted the role of self-efficacy in academic motivation. When students harbor negative beliefs about their academic capabilities, they are less likely to persist in the face of challenges, leading to disengagement and reduced motivation. Negative self-perceptions can create a cycle of self-doubt, where students fear failure and avoid taking on academic tasks, further contributing to diminished motivation [16].

High levels of stress resulting from these factors can lead to a reduction in students’ psychological resources, making them less able to manage the demands of their academic environment [48]. This can ultimately undermine their confidence and willingness to engage in academic tasks, causing a decline in motivation. Additionally, as research by Dweck [49] suggests, individuals who believe that their abilities are fixed and unchangeable tend to have lower motivation levels and are more likely to give up when faced with challenges. This highlights the importance of fostering positive academic self-perceptions and realistic academic expectations to buffer against the harmful effects of stress.

### Implications for practice

The findings of this study offer several important implications for educators and educational institutions. Educators can leverage these insights to design interventions that encourage students to adopt more adaptive emotion regulation strategies, like cognitive reappraisal, which involves reinterpreting negative situations in a more positive light. These strategies can empower students to better cope with academic stress and enhance their motivation. Additionally, creating an environment where students feel comfortable expressing their emotions and seeking support may reduce their reliance on suppression and ultimately lead to improved academic outcomes.

### Limitations and future directions

First, the study’s cross-sectional design limits the ability to draw conclusions about causality. Future research could adopt a longitudinal design to track changes in emotion regulation and academic stress over time and assess their impact on students’ academic motivation and performance more accurately.

Second, the study relied on self-report measures, which can be subject to response biases, such as social desirability or inaccurate self-assessment. Participants may have underreported stress levels or overestimated their ability to regulate their emotions. Future studies could incorporate multiple data sources, such as behavioral observations or peer/teacher assessments, to complement self-reported data and provide a more nuanced understanding of the factors influencing academic motivation.

Third, the study was conducted within a single cultural context, specifically in universities in China. The findings may not be directly generalizable to students in other countries or educational systems. To enhance the external validity of these findings, future research should replicate this study in different cultural and educational settings, examining whether the relationships between emotion regulation, academic stress, and motivation hold across diverse student populations.

In addition, this study uses convenience sampling through WeChat, without stratified procedures. While this approach facilitated timely data collection from diverse institutions, it may introduce self-selection bias and limit the generalizability of the results. Future research is recommended to employ stratified or randomized sampling techniques to ensure more representative samples and enhance the external validity of findings.

Furthermore, although we conducted multiple regression analyses to examine the predictive relationships among constructs, we did not conduct mediation or full structural equation modeling (SEM), which may have offered a more integrated understanding of the underlying mechanisms and theoretical pathways. Future research is encouraged to adopt longitudinal or SEM-based approaches to explore potential mediating or reciprocal effects among the variables.

## Conclusion

In conclusion, this study underscores the significant roles that both emotion regulation and perceptions of academic stress play in shaping students’ academic motivation. The findings reveal that emotion regulation, particularly the use of cognitive reappraisal, is a strong predictor of academic motivation. By helping students reframe negative situations in a more positive light, cognitive reappraisal fosters persistence, engagement, and a proactive approach to learning. On the other hand, perceptions of academic stress, particularly stress stemming from unrealistic academic expectations and negative self-perceptions, have a detrimental effect on students’ academic motivations. Importantly, the findings of this study call attention to the need for a holistic approach in promoting academic success. It is not enough to focus solely on academic skills or intellectual capabilities; psychological and environmental factors, such as emotional regulation and stress levels, must also be considered. Educators and institutions can play a pivotal role by creating environments that support emotional well-being and manage academic stress effectively, thereby fostering a more positive and motivating academic experience for students. These insights contribute to the growing body of research on student motivation and stress management, offering valuable guidance for educational practice. Future research could explore specific strategies for implementing interventions across diverse student populations and academic settings. By integrating emotion regulation and stress reduction techniques into educational frameworks, institutions can better support students in achieving their academic goals, while also promoting their psychological well-being.

## Supporting information

S1 Appendix Questionnaire. (DOCX)

## References

[1] Gass SM. Input, interaction, and the second language learner. Routledge. 2013.

[2] Ellis R. Understanding second language acquisition. 2nd ed. Oxford University Press. 2015.

[3] Loewen S. Introduction to instructed second language acquisition. Routledge. 2020.

[4] Anderman EM, Dawson H. Learning and motivation. In: Alexander PA, Mayer R. Handbook of Research on Learning and Instruction. Routledge. 2011. 219–41.

[5] Wentzel KR, Wigfield A. Introduction. Handbook of Motivation at School. Routledge. 2009. 1–8.

[6] Masgoret AM, Gardner RC. Attitudes, motivation, and second language learning: A meta‐analysis of studies conducted by Gardner and associates. Lang Learn. 2003;53(S1):167–210.

[7] Dörnyei Z, Schmidt R, Schmidt RW. Motivation and second language acquisition. Dörnyei Z, Schmidt R, Schmidt RW. Natl Foreign Lg Resource Ctr. 2001.

[8] Liu H, Wang Y, Wang H. Exploring the mediating roles of motivation and boredom in basic psychological needs and behavioural engagement in English learning: a self-determination theory perspective. BMC Psychol. 2025;13(1):179. doi: 10.1186/s40359-025-02524-3 40022265 PMC11871695

[9] Wang X, Feng Y, Xu Y, Liu H. Do Residential Areas Matter? Exploring the Differences in Motivational Factors, Motivation, and Learning Behaviors Among Urban High School English Learners From Different Regions. Education and Urban Society. 2025;57(4):347–68. doi: 10.1177/00131245251314194

[10] Wang X, Liu H. Exploring the Moderating Roles of Emotions, Attitudes, Environment, and Teachers in the Impact of Motivation on Learning Behaviours in Students’ English Learning. Psychol Rep. 2024;:332941241231714. doi: 10.1177/00332941241231714 38305018

[11] Noels KA, Pelletier LG, Clément R, Vallerand RJ. Why are you learning a second language? Motivational orientations and self‐determination theory. Lang Learn. 2003;53(S1):33–64.

[12] Abdolrezapour P, Jahanbakhsh Ganjeh S, Ghanbari N. Self-efficacy and resilience as predictors of students’ academic motivation in online education. PLoS One. 2023;18(5):e0285984. doi: 10.1371/journal.pone.0285984 37220147 PMC10204980

[13] Gross JJ, John OP. Individual differences in two emotion regulation processes: implications for affect, relationships, and well-being. J Pers Soc Psychol. 2003;85(2):348–62. doi: 10.1037/0022-3514.85.2.348 12916575

[14] Gross JJ. Emotion Regulation: Current Status and Future Prospects. Psychological Inquiry. 2015;26(1):1–26. doi: 10.1080/1047840x.2014.940781

[15] Gao Z, Yang Y. The predictive effect of trait emotional intelligence on emotion regulation strategies: The mediating role of negative emotion intensity. Syst. 2023;112:102958.

[16] Ryan RM, Deci EL. Self-determination theory and the facilitation of intrinsic motivation, social development, and well-being. Am Psychol. 2000;55(1):68–78. doi: 10.1037//0003-066x.55.1.68 11392867

[17] Gross JJ. Emotion regulation: affective, cognitive, and social consequences. Psychophysiology. 2002;39(3):281–91. doi: 10.1017/s0048577201393198 12212647

[18] Fredrickson BL. The role of positive emotions in positive psychology. The broaden-and-build theory of positive emotions. Am Psychol. 2001;56(3):218–26. doi: 10.1037//0003-066x.56.3.218 11315248 PMC3122271

[19] Csizér K, Dörnyei Z. The internal structure of language learning motivation and its relationship with language choice and learning effort. The Modern Language Journal. 2005 Mar;89(1):19–36.

[20] MacIntyre P, Gregersen T. Affect: The role of language anxiety and other emotions in language learning. Psychology for language learning: Insights from research, theory and practice. London: Palgrave Macmillan UK. 2012. 103–18.

[21] Rentzios C, Karagiannopoulou E, Ntritsos G. Academic Emotions, Emotion Regulation, Academic Motivation, and Approaches to Learning: A Person-Centered Approach. Behav Sci (Basel). 2025;15(7):900. doi: 10.3390/bs15070900 40723684 PMC12292873

[22] Teimouri Y, Plonsky L, Tabandeh F. L2 grit: Passion and perseverance for second-language learning. Lang Teach Res. 2022;26(5):893–918.

[23] Tharaldsen KB, Tvedt MS, Caravita SCS, Bru E. Academic stress: Links with emotional problems and motivational climate among upper secondary school students. Scand J Educ Res. 2023;67(7):1137–50.

[24] Walburg V. Burnout among high school students: A literature review. Child Youth Serv Rev. 2014;42:28–33.

[25] Lin S-H, Huang Y-C. Life stress and academic burnout. Active Learning in Higher Education. 2013;15(1):77–90. doi: 10.1177/1469787413514651

[26] Akanpaadgi E, Binpimbu F, Kuuyelleh EN. The impact of stress on students’ academic performance. Eureka: Journal of Educational Research. 2023;2(1):60–7.

[27] Weidner G, Kohlmann CW, Dotzauer E, Burns LR. The effects of academic stress on health behaviors in young adults. Anxiety, Stress, and Coping. 1996;9(2):123–33.

[28] MacGeorge EL, Samter W, Gillihan SJ. Academic stress, supportive communication, and health. Communication Education. 2005;54(4):365–72.

[29] Maqsood A, Gul S, Noureen N, Yaswi A. Dynamics of Perceived Stress, Stress Appraisal, and Coping Strategies in an Evolving Educational Landscape. Behav Sci (Basel). 2024;14(7):532. doi: 10.3390/bs14070532 39062355 PMC11274181

[30] Travis J, Kaszycki A, Geden M, Bunde J. Some stress is good stress: The challenge-hindrance framework, academic self-efficacy, and academic outcomes. J Educ Psychol. 2020;112(8):1632.

[31] Deci EL, Ryan RM. Intrinsic motivation and self-determination in human behavior. Plenum Publishing Co. 1985.

[32] Deci EL, Ryan RM. The “what” and “why” of goal pursuits: Human needs and the self-determination of behavior. Psychol Inq. 2000;11(4):227–68.

[33] Bielak J, Mystkowska‐Wiertelak A. Emotions and emotion regulation in L2 classroom speaking tasks: A mixed‐methods study combining the idiodynamic and quantitative perspectives. The Modern Language Journal. 2024;108(3):688–718.

[34] Bedewy D, Gabriel A. Examining perceptions of academic stress and its sources among university students: The Perception of Academic Stress Scale. Health Psychol Open. 2015;2(2):2055102915596714. doi: 10.1177/2055102915596714 28070363 PMC5193280

[35] Vallerand RJ, Pelletier LG, Blais MR. The Academic Motivation Scale: A measure of intrinsic, extrinsic, and amotivation in education. Educ Psychol Meas. 1992;52(4):1003–17.

[36] Orsini C, Binnie V, Evans P, Ledezma P, Fuentes F, Villegas MJ. Psychometric Validation of the Academic Motivation Scale in a Dental Student Sample. J Dent Educ. 2015;79(8):971–81. 26246537

[37] Pascual-Mariño J, Morales-García M, Sairitupa-Sanchez LZ, Mamani-Benito O, Mamani PGR, Morales-García SB, et al. Psychometric Properties of a Short Academic Motivation Scale (SAMS) in Medical Students. Behav Sci (Basel). 2024;14(4):316. doi: 10.3390/bs14040316 38667112 PMC11047349

[38] Fornell C, Larcker DF. Evaluating structural equation models with unobservable variables and measurement error. Journal of Marketing Research. 1981;18(1):39–50.

[39] Ben-Eliyahu A, Linnenbrink-Garcia L. Integrating the regulation of affect, behavior, and cognition into self-regulated learning paradigms among secondary and post-secondary students. Metacognition Learn. 2015;10(1):15–42.

[40] Zheng S, Zhou X. Positive Influence of Cooperative Learning and Emotion Regulation on EFL Learners’ Foreign Language Enjoyment. Int J Environ Res Public Health. 2022;19(19):12604. doi: 10.3390/ijerph191912604 36231905 PMC9564612

[41] Pekrun R. The control-value theory of achievement emotions: Assumptions, corollaries, and implications for educational research and practice. Educ Psychol Rev. 2006;18(4):315–41.

[42] Kwon K, Kupzyk K, Benton A. Negative emotionality, emotion regulation, and achievement: Cross-lagged relations and mediation of academic engagement. Learn Individ Differ. 2018;67:33–40.

[43] Tyra AT, Fergus TA, Ginty AT. Emotion suppression and acute physiological responses to stress in healthy populations: a quantitative review of experimental and correlational investigations. Health Psychol Rev. 2024;18(2):396–420. doi: 10.1080/17437199.2023.2251559 37648224 PMC12312699

[44] Lonigro A, Longobardi E, Laghi F. The interplay between expressive suppression, emotional self-efficacy and internalizing behavior in middle adolescence. Child Youth Care Forum. 2023;52(1):253–65.

[45] Lazarus RS, Folkman S. Stress, appraisal, and coping. Springer Publishing Company. 1984.

[46] Pekrun R, Goetz T, Titz W, Perry RP. Academic emotions in students’ self-regulated learning and achievement: A program of qualitative and quantitative research. Educ Psychol. 2002;37(2):91–105.

[47] Bandura A. Self-efficacy: The exercise of control. W. H. Freeman. 1997.

[48] Schunk DH, Zimmerman BJ. Motivation and self-regulated learning: Theory, research, and applications. Routledge. 2012.

[49] Dweck CS. Mindset: The new psychology of success. Random House. 2006.

