# Supplementary material for: Emotion regulation and perceptions of academic stress as key predictors of academic motivation in second language learning
Source: PLoS One. 2025 Aug 18;20(8):e0327071. doi: 10.1371/journal.pone.0327071 (PMC12360526; doi:10.1371/journal.pone.0327071)
Supplement: S1 Appendix — (DOCX) [file pone.0327071.s001.docx]

**Questionnaire**

**Basic Information**

- Age:
- Grade:
- Major:
- CET-4 Score (if applicable):
- CET-6 Score (if applicable):

**Emotion Regulation Questionnaire (ERQ)**

Please indicate your agreement with the following statements: 1 = Strongly Disagree | 2 = Disagree | 3 = Neutral | 4 = Agree | 5 = Strongly Agree

1. ____ When I want to feel more positive emotion (such as joy or amusement), I change what I’m thinking about.
2. ____ I keep my emotions to myself.
3. ____ When I want to feel less negative emotion (such as sadness or anger), I change what I’m thinking about.
4. ____ When I am feeling positive emotions, I am careful not to express them.
5. ____ When I’m faced with a stressful situation, I make myself think about it in a way that helps me stay calm.
6. ____ I control my emotions by not expressing them.
7. ____ When I want to feel more positive emotion, I change the way I’m thinking about the situation.
8. ____ I control my emotions by changing the way I think about the situation I’m in.
9. ____ When I am feeling negative emotions, I make sure not to express them.
10. ____ When I want to feel less negative emotion, I change the way I’m thinking about the situation.

**Perception of Academic Stress (PAS) Scale**

Please rate your perception of the following statements contributing to academic stress. Use the scale below: 1 = Strongly Disagree | 2 = Disagree | 3 = Neutral | 4 = Agree | 5 = Strongly Agree

1. ____ I am confident that I will be a successful student.
2. ____ I am confident that I will be successful in my future career.
3. ____ I can make academic decisions easily.
4. ____ The time allocated to classes and academic work is enough.
5. ____ I have enough time to relax after work.
6. ____ My teachers are critical of my academic performance.
7. ____ Teachers have unrealistic expectations of me.
8. ____ The unrealistic expectations of my parents stress me out.
9. ____ Competition with my peers for grades is quite intense.
10. ____ The size of the curriculum (workload) is excessive.
11. ____ I believe that the amount of work assignments is too much.
12. ____ I am unable to catch up if I get behind on work.
13. ____ The examination questions are usually difficult.
14. ____ Examination time is too short to complete the answers.
15. ____ Examination times are very stressful for me.
16. ____ I fear failing courses this year.
17. ____ I think that my worry about examinations is a weakness of character.
18. ____ Even if I pass my exams, I worry about getting a job.

**Academic Motivation Scale**

Using the scale below, indicate to what extent each of the following items corresponds to your reasons for learning English:
1 = Does not correspond at all
2 = Corresponds a little
3 = Corresponds moderately
4 = Corresponds a lot
5 = Corresponds exactly

**Items:**

1. ____ Because learning English will help me find a high-paying job in the future.
2. ____ Because I experience pleasure and satisfaction while improving my English skills.
3. ____ Because I think learning English will help me better prepare for the career I have chosen.
4. ____ For the intense feelings I experience when I successfully communicate my ideas in English.
5. ____ Honestly, I don't know; I really feel that I am wasting my time learning English.
6. ____ For the pleasure I experience while surpassing myself in learning English.
7. ____ To prove to myself that I am capable of mastering English.
8. ____ In order to obtain a more prestigious job that requires English proficiency.
9. ____ For the pleasure I experience when I discover new things in English that I never knew before.
10. ____ Because learning English will eventually enable me to work in a field that I like.
11. ____ For the pleasure that I experience when I read interesting materials in English.
12. ____ I once had good reasons for learning English; however, now I wonder whether I should continue.
13. ____ For the pleasure that I experience while I am surpassing myself in learning English as a personal accomplishment.
14. ____ Because of the fact that when I succeed in learning English, I feel important.
15. ____ Because I want to have "the good life" that English proficiency can bring.
16. ____ For the pleasure that I experience in broadening my knowledge about topics available in English.
17. ____ Because this will help me make better choices regarding my career goals.
18. ____ For the pleasure that I experience when I feel completely absorbed by what certain English writers have written.
19. ____ I can't see why I am learning English and frankly, I couldn't care less.
20. ____ For the satisfaction I feel when I am accomplishing difficult tasks in learning English.
21. ____ To show myself that I am an intelligent person capable of mastering English.
22. ____ In order to have a better salary that English skills can help me achieve.
23. ____ Because studying English allows me to continue learning about many topics that interest me.
24. ____ Because I believe that learning English will improve my overall competence in the job market.
25. ____ For the "high" feeling that I experience while engaging with interesting English materials.
26. ____ I don't know; I can't understand why I am learning English.
27. ____ Because mastering English gives me a personal satisfaction in my quest for excellence.
28. ____ Because I want to show myself that I can succeed in learning English.
